# Supplementary material for: Identification of Genes Associated with Gut Microbiota Metabolites in Type 2 Diabetes Mellitus Complicated by Acute Cerebral Infarction Based on Bioinformatics and Mendelian Randomization
Source: Biomedicines. 2026 Oct 17;14(10):2371. doi: 10.3390/biomedicines14102371 (PMC13702449; doi:10.3390/biomedicines14102371)
Supplement: Supplementary file 1 [file biomedicines-14-02371-s001.zip › Figure S2/genus.Flavonifractor.id.2059 T2DM.pdf]

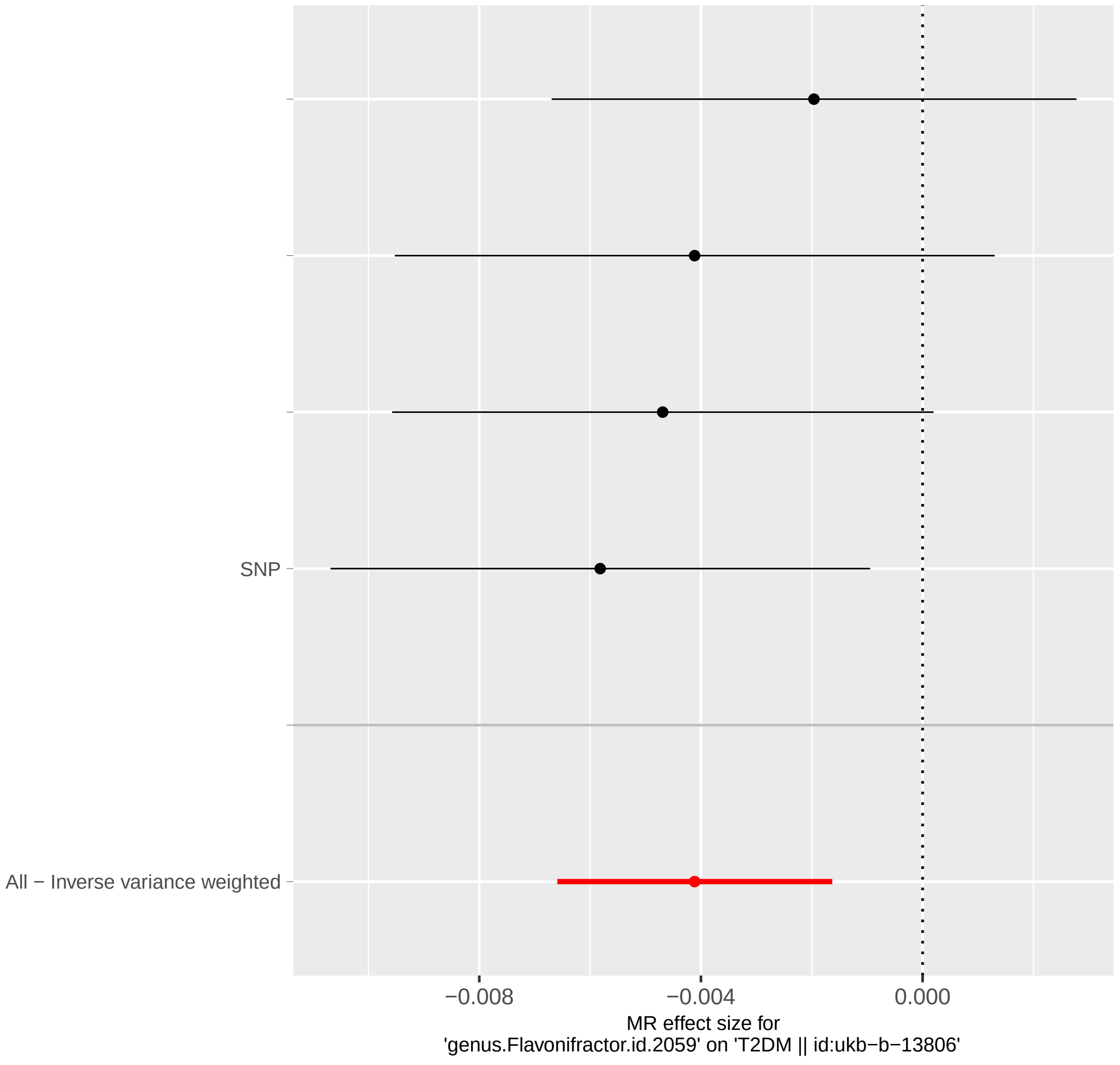

SNP
All − Inverse variance weighted
−0.008
−0.004
0.000
MR effect size for
'genus.Flavonifractor.id.2059' on 'T2DM || id:ukb−b−13806'
